# Embryonic and Postnatal Expression of Aryl Hydrocarbon Receptor mRNA in Mouse Brain

**DOI:** 10.3389/fnana.2017.00004

**Published:** 2017-02-07

**Authors:** Eiki Kimura, Chiharu Tohyama

**Affiliations:** ^1^Laboratory of Environmental Health Sciences, Center for Disease Biology and Integrative Medicine, Graduate School of Medicine, The University of TokyoTokyo, Japan; ^2^Environmental Biology Laboratory, Faculty of Medicine, University of TsukubaTsukuba, Japan

**Keywords:** aryl hydrocarbon receptor, cerebellum, cerebral cortex, hippocampus, *in situ* hybridization, mouse, olfactory bulb, rostral migratory stream

## Abstract

Aryl hydrocarbon receptor (AhR), a member of the basic helix-loop-helix-Per-Arnt-Sim transcription factor family, plays a critical role in the developing nervous system of invertebrates and vertebrates. Dioxin, a ubiquitous environmental pollutant, avidly binds to this receptor, and maternal exposure to dioxin has been shown to impair higher brain functions and dendritic morphogenesis, possibly via an AhR-dependent mechanism. However, there is little information on AhR expression in the developing mammalian brain. To address this issue, the present study analyzed AhR mRNA expression in the brains of embryonic, juvenile, and adult mice by reverse transcription (RT)-PCR and *in situ* hybridization. In early brain development (embryonic day 12.5), AhR transcript was detected in the innermost cortical layer. The mRNA was also expressed in the hippocampus, cerebral cortex, cerebellum, olfactory bulb, and rostral migratory stream on embryonic day 18.5, postnatal days 3, 7, and 14, and in 12-week-old (adult) mice. Hippocampal expression was abundant in the CA1 and CA3 pyramidal and dentate gyrus granule cell layers, where expression level of AhR mRNA in 12-week old is higher than that in 7-day old. These results reveal temporal and spatial patterns of AhR mRNA expression in the mouse brain, providing the information that may contribute to the elucidation of the physiologic and toxicologic significance of AhR in the developing brain.

## Introduction

The aryl hydrocarbon receptor (AhR) is a ligand-activated transcription factor belonging to the basic helix-loop-helix (bHLH)-Per-Arnt-Sim (PAS) family that is expressed in various organs. Its activation induces downstream pathways via expression of AhR-target genes such as *cytochrome P450* (*Cyp*)*1a1* and *Cyp1b1* (Furness and Whelan, 2009). The gene encoding AhR is evolutionarily conserved from *Caenorhabditis elegans* to mammals (Hahn, 2002), and is thought to play essential roles in developmental and physiological processes. The function of AhR in the developing nervous system has been investigated by loss- and gain-of-function experiments. *C. elegans* *ahr-1* was shown to regulate neuronal subtype specification, cell migration, and axonal branching (Huang et al., 2004; Qin and Powell-Coffman, 2004), while the *Drosophila* homolog *Spineless* controls dendritic arborization in sensory neuron subtypes (Kim et al., 2006). AhR deficiency in cerebellar neuron precursors during development leads to impairment of neurogenesis in mice (Dever et al., 2016). These results suggest an important role of AhR in nervous system development. In fact, bHLH transcription factors are known to regulate developmental processes of the mammalian brain: in the cerebral cortex, Hairy and enhancer of split (Hes)1 and Hes5 inhibit neuronal differentiation to maintain the neural stem cell pool (Ishibashi et al., 1994; Ohtsuka et al., 2001), while *c*-Myc and *N*-Myc regulate cellular proliferation in the cerebral cortex and cerebellum (Wey and Knoepfler, 2010; Wey et al., 2010). In addition, Single-minded homolog (Sim)1 and Sim2 have been implicated in neuronal subtype specification in the hypothalamus (Michaud et al., 1998; Goshu et al., 2004). Similarly, AhR may regulate developmental processes in various brain regions. Although a previous study (Petersen et al., 2000) reported the presence of AhR mRNA in various brain regions of adult rats, no report has been available in the embryonic and early postnatal stages of *in situ* the developing mammalian brain.

Dioxin is a ubiquitous environmental contaminant and a known exogenous AhR ligand that has carcinogenic effects as well as reproductive and immune toxicity (Mandal, 2005). The majority of these toxic effects are exerted via an AhR-dependent mechanism, as revealed by studies using AhR-null mice (Fernandez-Salguero et al., 1995; Schmidt et al., 1996; Mimura et al., 1997). In addition, rodents born to dams exposed to dioxin exhibit cognitive and behavioral abnormalities, including loss of behavioral flexibility, paired associate learning and memory, anxiety, and social behavior (Schantz et al., 1996; Markowski et al., 2001; Hojo et al., 2002; Mitsui et al., 2006; Haijima et al., 2010; Endo et al., 2012; Kakeyama et al., 2014). We recently found that exposure to dioxin *in utero* or through lactation causes abnormalities in dendritic morphology and upregulates the expression of class 3 semaphorin genes in the developing mouse brain (Kimura et al., 2015, 2016). These results suggest that the dioxin-AhR complex disrupts critical processes during brain development that ensure higher brain function in adulthood. Therefore, it would be worth studying spacio-temporal expression of AhR in the developing brain for understanding not only its possible physiologic roles, but also the mechanism of developmental neurotoxicity of dioxin.

To address this issue, the present study was performed to examine AhR mRNA expression in the developing mouse brain using reverse transcription (RT)-PCR method and a novel *in situ* hybridization approach known as RNAscope (Wang et al., 2012). We selected the hippocampus, cerebral cortex, cerebellum, and olfactory bulb for analysis, since perinatal dioxin exposure was previously found to induce AhR target gene expression in these regions (Kimura et al., 2016). We show the histochemical localization of AhR mRNA in the various regions of mouse brains during embryonic, juvenile, and adult periods.

## Materials and Methods

### Reagents

Reagents were of analytical grade and purchased from Wako Pure Chemicals (Osaka, Japan), unless otherwise stated.

### Animals

Experimental protocols were approved by the Animal Care and Use Committee of the University of Tokyo. Pregnant female or 12-week-old male C57BL/6J mice were purchased from CLEA Japan (Tokyo, Japan) and housed in an animal facility at a temperature of 22°C–24°C and humidity of 40–60% on a 12:12-h light/dark cycle (lights on from 08:00 to 20:00). Laboratory rodent chow (Lab MR Stock; Nosan, Yokohama, Japan) and distilled water were provided *ad libitum*. Male offspring at embryonic days (EDs) 12.5 or 18.5, or postnatal days (PNDs) 3, 7, or 14 were selected for *in situ* hybridization analysis (*n* = 3 mice at each stage of growth).

### Genotyping PCR

To examine the sex of mouse embryos, genomic DNA was extracted from tail tips by lysing in 50 mM NaOH at 95°C for 2 h. The lysate was mixed with 1 M Tris-HCl (pH 8.0) for neutralization, and centrifuged at 12,000 rpm at room temperature for 10 min. The genomic DNA in the supernatant was used as the template for PCR using a Takara Ex Taq PCR kit (Takara Bio, Kusatsu, Japan) on a Veriti thermal cycler (Applied Biosystems, Foster City, CA, USA). The amplification conditions were as follows: 95°C for 1 min; and 40 cycles of 95°C for 15 s, 60°C for 30 s, and 72°C for 30 s. The PCR primers for amplifying the murine Sex-determining region Y (*Sry*) gene were 5′-gtggtgagaggcacaagttggc-3′ and 5′-ctgtgtaggatcttcaatctct-3′. The 20-μl reaction contained 250 nM each primer, 1 × Ex Taq reaction buffer, 200 μM each deoxynucleoside triphosphate (dNTP mixture), and 0.5 U of Ex Taq DNA polymerase. PCR products were separated by electrophoresis on agarose gels that were stained with ethidium bromide. Genomic DNA from male embryos showed PCR products of the expected size (140 bp).

### RT-PCR

Male offspring were sacrificed by decapitation on PND 7, and brain regions, including the hippocampus, cerebral cortex, cerebellum, and olfactory bulb, were quickly separated, and stored at -80°C until analysis by RT-PCR. The total RNA was isolated from the each brain region using an RNeasy Mini Kit (Qiagen, Tokyo, Japan). The cDNA for a given mRNA was synthesized using oligo-dT and random hexamers with a Primescript RT reagent kit (Takara Bio). Expression of AhR and GAPDH mRNA were determined using Veriti thermal cycler (Applied Biosystems) with KOD Plus kit (Toyobo, Osaka, Japan). The amplification conditions were as follows: 95°C for 1 min; and 30 cycles of 95°C for 15 s, 55°C for 15 s, and 68°C for 15 s. The PCR primers for amplifying the murine AhR and GAPDH mRNA were 5′-tgtagagcacaaatcagaga-3′/5′-gatagtggaggaagcatag-3′, and 5′-acccagaagactgtggatgg-3′/5′-cacattgggggtaggaacac-3′, respectively. The 25-μl reaction solution contained 250 nM each primer, 1 × KOD Plus buffer, 200 μM dNTP mixture, 1 mM MgSO_4_, and 0.5 U of KOD Plus DNA polymerase. PCR products were separated by electrophoresis on agarose gels that were stained with Midori Green Advance (Nippon Gene, Tokyo, Japan). PCR products of AhR and GAPDH mRNA were expected to have 123 and 171 bp in size, respectively.

### Tissue Preparation

Whole ED 12.5 embryos and ED 18.5 brains were fixed overnight with 4% paraformaldehyde (PFA) in 0.1 M phosphate-buffered saline (PBS, pH 7.4). Male mice that were 3, 7, or 14 days or 12 weeks old, were anesthetized with sodium pentobarbital and perfused transcardially with 4% PFA in 0.1 M PBS, and the brains were harvested and post-fixed overnight with 4% PFA. Whole embryos or brains were dehydrated through a graded ethanol series and xylene and embedded in paraffin. Sagittal sections were cut at a thickness of 5 μm using a sliding microtome (LS-113; Yamato Kohki, Saitama, Japan).

### *In situ* Hybridization

*In situ* hybridization was performed using the RNAscope 2.0 HD Reagent kit (Brown) (Advanced Cell Diagnostics, Hayward, CA, USA) according to the manufacturer’s instructions. Mouse AhR mRNA RNAscope probes were custom-made by Advanced Cell Diagnostics and targeted bases 867–1836 of mouse AhR mRNA (NCBI reference sequence: NM_013464.4). Probe sets specific for the housekeeping gene peptidylprolyl isomerase B (Ppib) from mouse and the dapB gene from *Bacillus subtilis* were used as positive and negative controls, respectively. After deparaffinization and dehydration, formalin-fixed sections were pretreated with protease and then subjected to *in situ* hybridization. Briefly, sections were hybridized with the probe solution, followed by amplification and probe detection using staining solutions. The sections were then stained with Gill’s hematoxylin to visualize individual cells in each brain region. Slides were covered with Marinol (Muto Chemicals, Tokyo, Japan) and a plastic coverslip before viewing with a Leica DM6000 B microscope (Leica Microsystems, Tokyo, Japan). Bright-field images were captured using a Leica DM6000 B microscope with specific objective lenses (HCX PL APO, 40 ×, NA = 0.75; Leica Microsystems).

### Punctate Signal Density Analysis

The number of brown punctate signals of AhR mRNA were counted in the pyramidal cell layer, stratum orience (SO), and stratum radiatum (SR) in the CA1 and CA3, and the granule cell layer and hilus in the DG of the hippocampus from 7-day-old and 12-week-old mice. Punctate signal density was calculated by dividing the punctate signal number by area of each brain subregion.

### Statistical Analysis

Expression level of AhR mRNA by RT-PCR was analyzed using the one-way analysis of various (ANOVA). Punctate density of AhR mRNA signals was analyzed using the two-way repeated measures ANOVA, followed by Tukey-Kramer test or Student’s *t*-test. *P*-values < 0.05 were considered statistically significant.

## Results

### RT-PCR Analysis of AhR mRNA Expression in the Developing Mouse Brain

**FIGURE 1:**
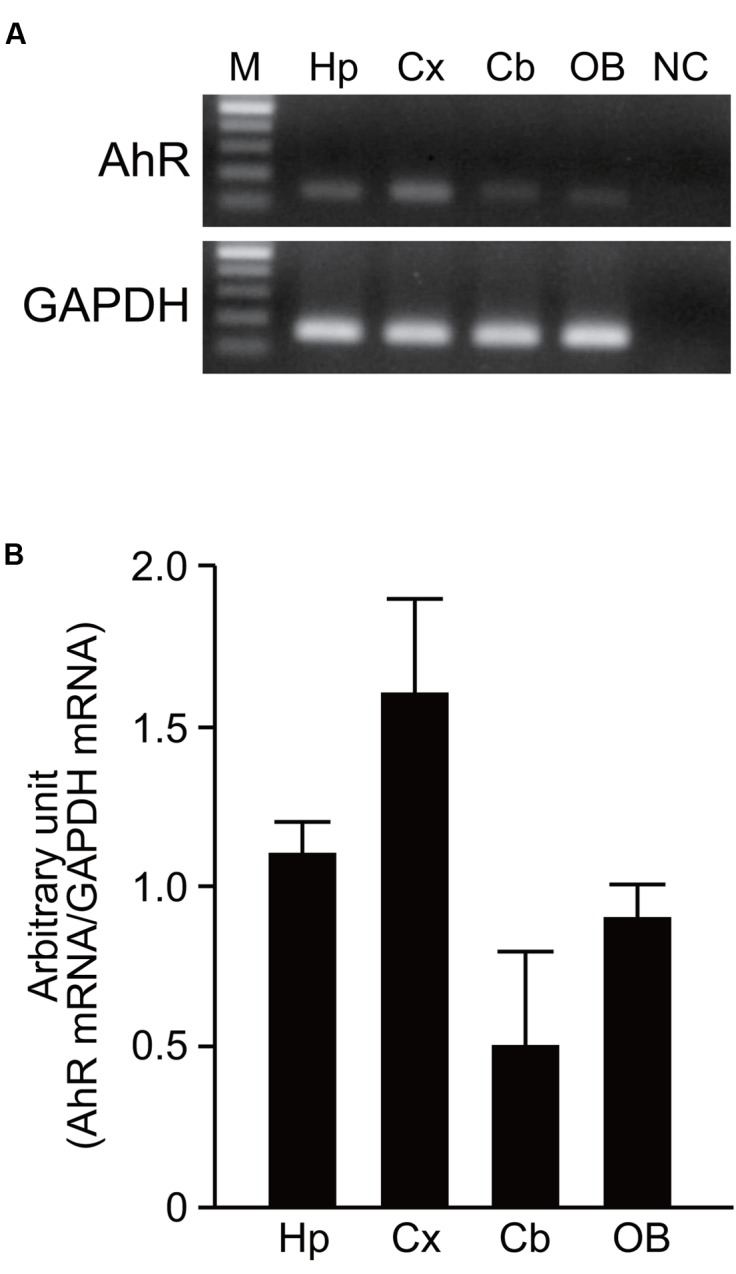
**AhR mRNA expression in the developing mouse brain. (A)** AhR and GAPDH mRNA were amplified by RT-PCR in the hippocampus, cerebral cortex, cerebellum, and olfactory bulb on PND 7. **(B)** Semi-quantitative analysis showed expression levels of AhR mRNA in four brain regions. The value was normalized by GAPDH mRNA expression. M, 100 bp marker; Hp, hippocampus; Cx, cerebral cortex; Cb, cerebellum; OB, olfactory bulb; NC, negative control. The values are shown as mean ± SEM for three mice/brain region.

AhR mRNA was detected in the hippocampus, cerebral cortex, cerebellum, and olfactory bulb of 7-day-old mice by RT-PCR analysis (**Figure 1A**). However, no significant difference in expression levels of AhR mRNA was observed in the four brain regions by semi-quantitative analysis (one-way ANOVA, *p* = 0.069; **Figure 1B**).

### Histological Analysis of AhR mRNA Expression in the Embryonic Mouse Brain

**FIGURE 2:**
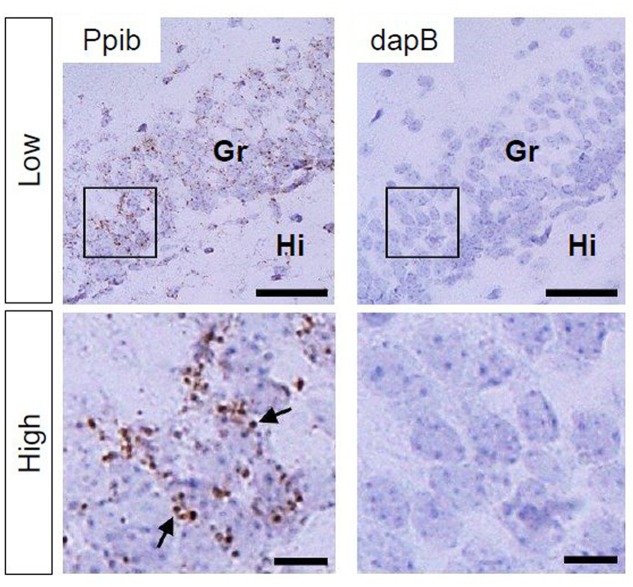
**Peptidylprolyl isomerase (Ppib) and dapB mRNA expression in the hippocampal DG in PND 14 mice.** Brown punctae (arrows) represent Ppib mRNA (left). No signal associated with dapB mRNA was observed (right). “Low” and “High” indicate magnification. High magnification images represent the areas enclosed by boxes in the low-magnification images. Scale bars = 50 and 10 μm in low and high magnification images, respectively. Gr, granule cell layer; Hi, hilus.

**FIGURE 3:**
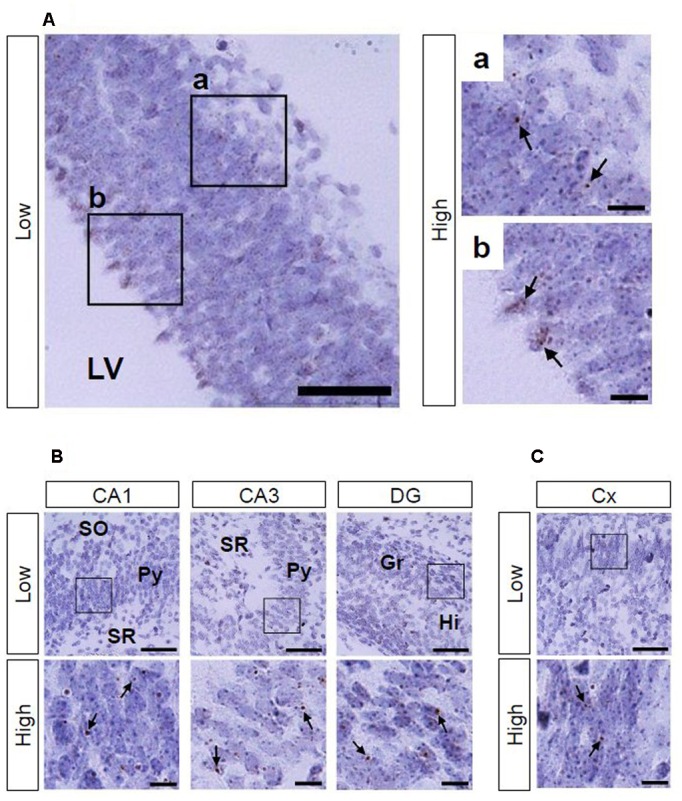
**AhR mRNA expression in the telencephalon of mice on ED 12.5 and ED 18.5.** Brown punctae (arrows) represent AhR transcript in the cerebral cortex **(A)** on ED 12.5, and in the hippocampus **(B)** and cerebral cortex **(C)** on ED 18.5. “Low” and “High” indicate magnification. High magnification images represent the areas enclosed by boxes in the low-magnification images. Scale bars = 50 and 10 μm in low and high magnification images, respectively. Gr, granule cell layer; Hi, hilus; LV, lateral ventricle; Py, pyramidal cell layer; SO, stratum oriens; SR, stratum radiatum.

Using Ppib mRNA and dapB mRNA as positive and negative controls, respectively, for *in situ* hybridization by the RNAscope, we confirmed that the former showed brown punctate, but that the latter did not show any signals under the present experimental conditions (**Figure 2**). We evaluated AhR mRNA levels in the brain on ED 12.5 and ED 18.5. AhR transcript was detected in the cerebral cortex on ED 12.5 (**Figure 3A**), with strong signals in the innermost cortical layer. On ED 18.5, signals were observed in the hippocampus and cerebral cortex (**Figures 3B,C**). In the hippocampus, AhR mRNA signals were detected in the pyramidal cell layer of the CA1 and CA3, and granule cell layer of the DG regions (**Figure 3B**).

### Histological Analysis of AhR mRNA Expression in the Juvenile Mouse Brain

**FIGURE 4:**
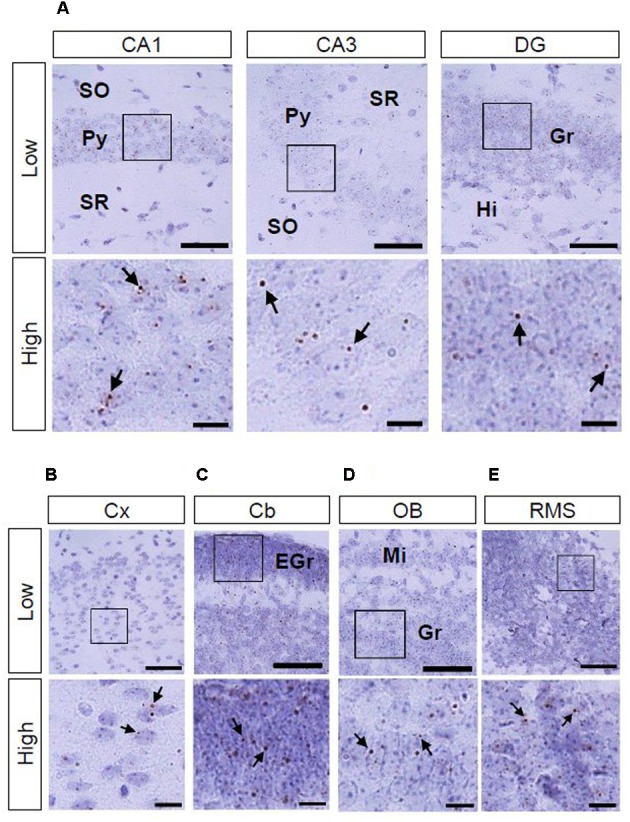
**AhR mRNA expression in various regions of mice on PND 7.** Brown punctae (arrows) represent AhR transcript in the hippocampus **(A)**, cerebral cortex **(B)**, cerebellum **(C)**, olfactory bulb **(D)**, and RMS **(E)**. “Low” and “High” indicate magnification. High magnification images represent the areas enclosed by boxes in the low-magnification images. Scale bars = 50 and 10 μm in low and high magnification images. EGr, external granule cell layer; Gr, granule cell layer; Hi, hilus; Mi, mitral cell layer; Py, pyramidal cell layer; SO, stratum oriens; SR, stratum radiatum.

We also investigated AhR mRNA expression in the mouse brain on PNDs 3, 7, and 14. The transcript was observed in the CA1 and CA3 pyramidal cell layers and DG granule cell layer of the hippocampus (**Figure 4A**; **Supplementary Figures S1A** and **S2A**) as well as in the cerebral cortex (**Figure 4B**; **Supplementary Figures S1B** and **S2B**) at all three time points. In the cerebellum, AhR mRNA signals were present in the external granule cell layer on PNDs 3 (**Supplementary Figure S1C**) and 7 (**Figure 4C**) and in the granule cell layer on PND 14 (**Supplementary Figure S2C**). AhR mRNA signals were also detected in the granule cell layer of the olfactory bulb (**Figure 4D**; **Supplementary Figures S1D** and **S2D**) and the rostral migratory stream (RMS) on PNDs 3, 7, and 14 (**Figure 4E**; **Supplementary Figures S1E** and **S2E**).

### Histological Analysis of AhR mRNA Expression in the Adult Mouse Brain

**FIGURE 5:**
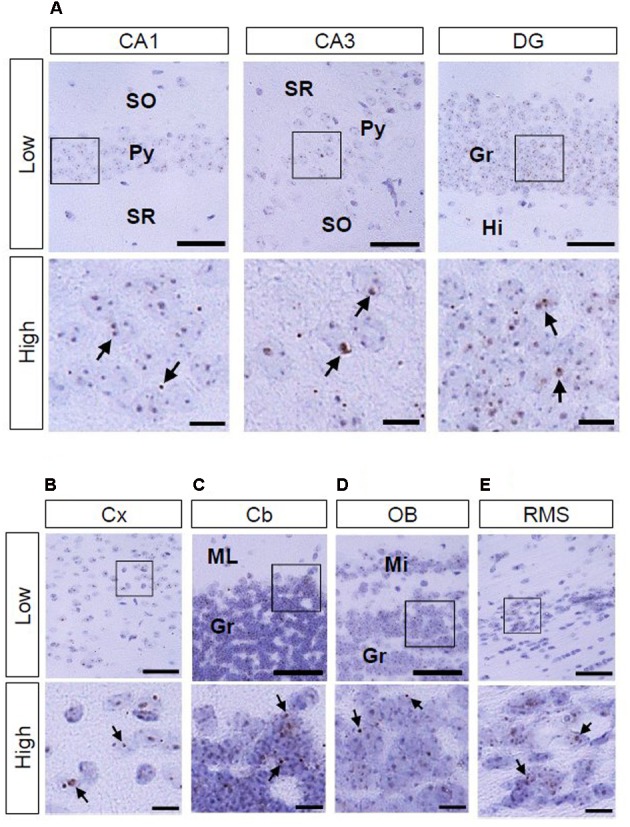
**AhR mRNA expression in various regions of mice in 12-week old.** Brown punctae (arrows) represent AhR transcript in the hippocampus **(A)**, cerebral cortex **(B)**, cerebellum **(C)**, olfactory bulb **(D)**, and RMS **(E)**. “Low” and “High” indicate magnification. High magnification images represent the area enclosed by boxes in the low-magnification images. Scale bars = 50 and 10 μm in low and high magnification images. Gr, granule cell layer; Hi, hilus; Mi, mitral cell layer; ML, molecular layer; Py, pyramidal cell layer; SO, stratum oriens; SR, stratum radiatum.

As to AhR mRNA expression in the brain of adult (12-week-old) mice, signals were observed in the hippocampal CA1 and CA3 pyramidal and DG granule cell layers, cerebral cortex, cerebellar and olfactory bulb granule cell layers, and RMS (**Figures 5A–E**).

### Quantitative Analysis of AhR mRNA Expression in the Developing and Adult Hippocampus

**FIGURE 6:**
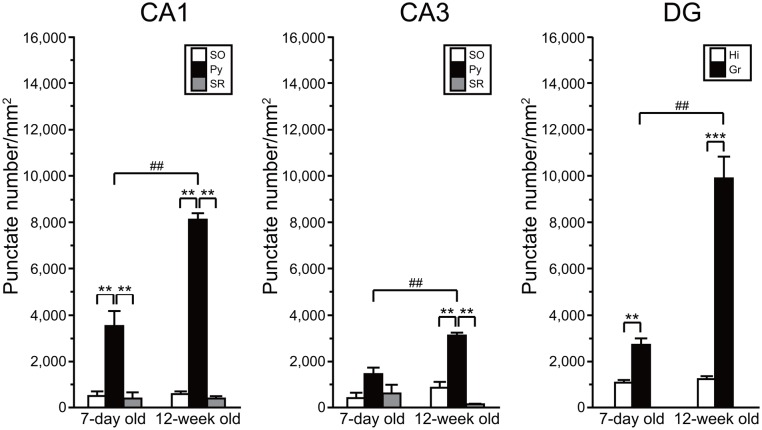
**Quantitative analysis of density of AhR mRNA signals in the hippocampal subregions in 7-day and 12-week old.** Punctate density of AhR mRNA signals in the CA1, CA3, and DG in the hippocampus. Signal density in the CA1 and CA3 pyramidal cell layer (Py) and DG granule cell layer (Gr) was dramatically higher than that in the stratum oriens (SO) and stratum radiatum (SR) of CA1 and CA3, and hilus (Hi) of DG in 7-day- and 12-week-old mice. Signal density in the Py and Gr in 12-week-old mice was significantly increased when compared with that in the 7-day-old mice. ^∗∗^ and ^∗∗∗^ indicate significant difference between the subregions (*p* < 0.01 and 0.001, respectively). ## indicates significant difference between ages (*p* < 0.01). The values are shown as mean ± SEM for three mice/age.

We compared the density of punctate signals of AhR mRNA between subregions of the hippocampus and between ages (7-day old vs. 12-week old). Two-way repeated measures ANOVA indicated significant differences in the number of AhR mRNA punctate signals by subregion and by age, and a significant interaction between these two factors in the CA1 (*p* = 6.33 × 10^-10^, 5.05 × 10^-5^, and 8.70 × 10^-6^, respectively), CA3 (*p* = 2.04 × 10^-5^, 1.25 × 10^-3^, and 1.56 × 10^-2^, respectively), and DG (*p* = 5.89 × 10^-6^, 6.67 × 10^-5^, and 9.24 × 10^-5^, respectively; **Figure 6**). In the hippocampal CA1 regions, the punctate signal density in the pyramidal cell layer was significantly higher than that in the SO and SR in 7-day-old mice (*p* < 0.01 and 0.01, respectively) and adult mice (*p* < 0.01 and 0.01, respectively). The signal density in the CA3 pyramidal cell layer was significantly higher than that in the SO and SR in adult mice (*p* < 0.01 and 0.01, respectively). Furthermore, the punctate signal density in the granule cell layer in DG was significantly higher than that in the hilus in 7-day-old mice and adult mice (*p* < 0.01 and 0.001, respectively). As to age, the punctate signal density in the CA1 and CA3 pyramidal and DG granule cell layers was significantly higher in the 12-week-old mice than that in the 7-day-old mice (*p* < 0.01, 0.01, and 0.01, respectively; **Figure 6**).

## Discussion

The present study using *in situ* hybridization demonstrated for the first time that AhR mRNA is expressed during embryonic development as well as postnatally in specific regions of the mouse brain. In development of the nervous system, an essential role of AhR has been demonstrated in a wide spectrum of animals, ranging from *C. elegans* to mouse. In brief, AhR regulates neuronal subtype specification, cellular migration, axonal branching and dendritic arborization in *C. elegans* and *Drosophila* (Huang et al., 2004; Qin and Powell-Coffman, 2004; Kim et al., 2006). In mice, neuronal progenitors lacking AhR show an impairment of neurogenesis in the developing cerebellum (Dever et al., 2016). In the present study, AhR mRNA was detected in the developing brain regions, including the hippocampus, cerebral cortex, cerebellum, and olfactory bulb (**Figure 1**). Hence, it is reasonable to consider that AhR has developmental and physiological roles in the mammalian brain.

In the present study, AhR mRNA was detected in the cerebral cortex on ED 12.5 (**Figure 3A**); importantly, the signal was strongest in the innermost cortical layer that is known to harbor neural stem cells in a previous study (Noctor et al., 2001). Thus, AhR may function in a manner similar to Hes1 and Hes5, which suppress differentiation of neural precursor cells (Ishibashi et al., 1994; Ohtsuka et al., 2001), and thereby control the timing and extent of neurogenesis in the cortex. AhR mRNA was expressed in the hippocampus, cerebral cortex, cerebellum, olfactory bulb, and RMS on PNDs 3 (**Supplementary Figure S1**), 7 (**Figure 4**), and 14 (**Supplementary Figure S2**). In the postnatal brain, dendritic and axonal elongation is essential for neural circuit formation, which is regulated by class 3 semaphorin genes (Polleux et al., 1998, 2000; Falk et al., 2005; Taniguchi et al., 2005; Takeuchi et al., 2010) as well as brain-derived neurotrophic factor (BDNF) (McAllister et al., 1995; Niblock et al., 2000). Glutamate and GABA receptor signaling also promotes dendritic arborization and synapse formation (Cline, 2001; Sernagor et al., 2010). Altered expression of genes by 2,3,7,8-teterachlorodibenzo-*p*-dioxin (TCDD), a most effective AhR agonist, has been reported. Class 3 semaphorin genes were induced by TCDD in the brain of red sea bream (*Pagrus major*) embryos and that of juvenile mice (Iida et al., 2013; Kimura et al., 2016). Gene expression of NMDA receptor subunit NR2A was enhanced, but NR2B was suppressed on PND 49 by *in utero* and lactational exposure to TCDD (Kakeyama et al., 2001). AhR knockdown reduced NMDA receptor subunit expression, attenuated NMDA receptor-mediated excitatory post-synaptic currents, and enhanced NMDA-induced BDNF expression (Lin et al., 2009). In addition, postnatal TCDD exposure reduces expression of GABA receptor subunit in the cerebellum (Collins et al., 2008). Expression of genes, such as neural cell adhesion molecule 1 and synaptophysin, which are involved in neural circuit formation in the embryonic telencephalon, was suppressed by TCDD exposure (Gohlke et al., 2009). Thus, AhR may participate in neural circuit formation by regulating gene expression.

*In situ* AhR mRNA expression has been reported in various regions of adult Sprague-Dawley rat brain, including the cerebral cortex, hippocampus, cerebellum, and olfactory bulb (Petersen et al., 2000). The transcript was also detected in neural stem cells in the subgranular zone of the hippocampal DG in adult C57BL/6 mice (Latchney et al., 2013). We observed AhR mRNA signals in the cerebral cortex; hippocampal CA1, CA3, and DG regions; cerebellum; and olfactory bulb in adult mice (**Figures 5A–D**), which is consistent with observations in adult rats (Petersen et al., 2000). AhR has been implicated in adult neurogenesis in the DG (Latchney et al., 2013). Besides, adult neurogenesis is observed in the granule neurons of the olfactory bulb in adulthood (Zhao et al., 2008). Neuronal precursors from the subventricular zone of the lateral ventricle migrate to the olfactory bulb via the RMS throughout adulthood. Our observation that AhR mRNA was expressed in the granule cell layer of the olfactory bulb and was present in the RMS in adult mice (**Figures 5D,E**) implies that AhR regulates adult neurogenesis not only in the hippocampus but also in the olfactory bulb. In the hippocampus, AhR transcript levels were elevated in the CA1 and CA3 pyramidal and DG granule cell layers (**Figure 6**) that are known to be rich in the number of neurons. In the CA1 and CA3 pyramidal and DG granule cell layers, expression levels of AhR mRNA in adult mice were significantly increased when compared with that in the developmental stage (**Figure 6**). AhR-null mice showed impairment of fear memory in adulthood (Latchney et al., 2013); thus, these results suggest that AhR expressed in neurons plays important roles not only in developmental processes, but also in higher brain function.

AhR mRNA expression was hardly observed in the fetal, postnatal, and adult periods of mice in the Allen Brain Atlas (ABA) (Lein et al., 2007; Henry and Hohmann, 2012). On the other hand, our present study successfully detected the AhR mRNA expression in multiple regions of the brain. The inconsistent results between our study and the ABA may be due to the following reasons. First, the detection sensitivity of the RNAscope may be much higher than the digoxygenin-labeled antisense probe used in the ABA. Second, the target regions of probes used are different between two studies. The present study and ABA used base sequences of 867–1836 and 3596–4276, respectively.

Perinatal dioxin exposure perturbs dendritic morphology of hippocampal CA1 pyramidal neurons (Kimura et al., 2015), cortical layer formation (Mitsuhashi et al., 2010), and neurogenesis in cerebellar granule cells (Collins et al., 2008). Although the molecular mechanisms underlying the developmental neurotoxicity of dioxin remain unclear, our observations suggest that dioxin causes hyperactivation of AhR and downstream signaling pathways in AhR-expressing cells in the brain, thereby dysregulating developmental processes required for higher brain function in adulthood. Adverse circumstances during gestational period have been reported to be a cause of adult onset diseases, and the paradigm named ‘developmental origins of health and disease’ has been widely accepted (Wadhwa et al., 2009). The present findings suggest that this theory can be applied to environmental chemical exposure during the developmental period of life.

## Author Contributions

EK and CT conceived and designed this study; EK performed experiments and analyzed data; EK and CT wrote the manuscript.

## Conflict of Interest Statement

The authors declare that the research was conducted in the absence of any commercial or financial relationships that could be construed as a potential conflict of interest.

## Supplementary Material

FIGURE S1 **AhR mRNA expression in various regions of mice on PND 3.** Brown punctae (arrows) represent AhR transcript in the hippocampus **(A)**, cerebral cortex **(B)**, cerebellum **(C)**, olfactory bulb **(D)**, and RMS **(E)**. “Low” and “High” indicate magnification. High magnification images represent the areas enclosed by boxes in the low-magnification images. Scale bars = 50 and 10 μm in low and high magnification images. EGr, external granule cell layer; Gr, granule cell layer; Hi, hilus; Mi, mitral cell layer; Py, pyramidal cell layer; SO, stratum oriens; SR, stratum radiatum.

FIGURE S2 **AhR mRNA expression in various regions of mice on PND 14.** Brown punctae (arrows) represent AhR transcript in the hippocampus **(A)**, cerebral cortex **(B)**, cerebellum **(C)**, olfactory bulb **(D)**, and RMS **(E)**. “Low” and “High” indicate magnification. High magnification images represent the areas enclosed by boxes in the low-magnification images. Scale bars = 50 and 10 μm in low and high magnification images. Gr, granule cell layer; Hi, hilus; Mi, mitral cell layer; ML, molecular layer; Py, pyramidal cell layer; SO, stratum oriens; SR, stratum radiatum.
