# Supplementary figures and images for: Embryonic and Postnatal Expression of Aryl Hydrocarbon Receptor mRNA in Mouse Brain
Source: Front Neuroanat. 2017 Feb 7;11:4. doi: 10.3389/fnana.2017.00004 (PMC5293765; doi:10.3389/fnana.2017.00004)

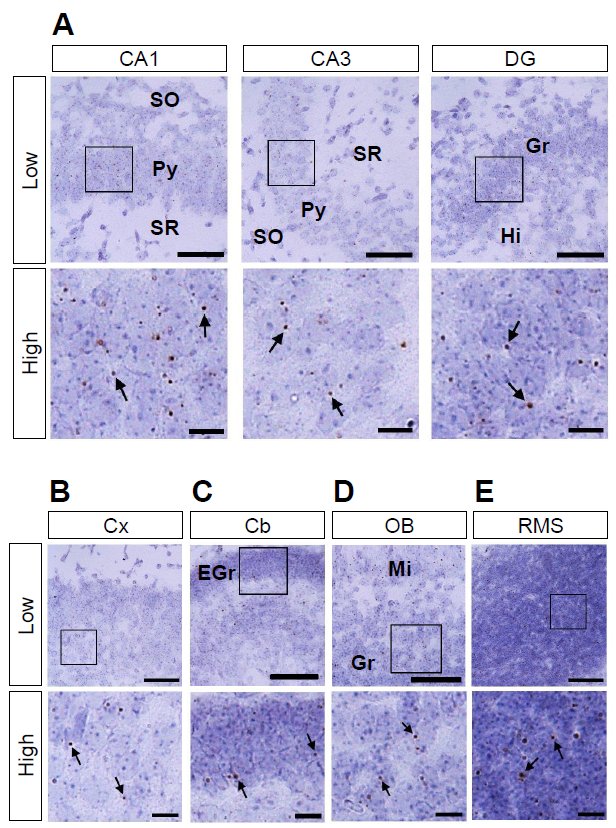

Supplement: FIGURE S1 — AhR mRNA expression in various regions of mice on PND 3. Brown punctae (arrows) represent AhR transcript in the hippocampus (A), cerebral cortex (B), cerebellum (C), olfactory bulb (D), and RMS (E). “Low” and “High” indicate magnification. High magnification images represent the areas enclosed by boxes in the low-magnification images. Scale bars = 50 and 10 μm in low and high magnification images. EGr, external granule cell layer; Gr, granule cell layer; Hi, hilus; Mi, mitral cell layer; Py, pyramidal cell layer; SO, stratum oriens; SR, stratum radiatum. [file Image_1.JPEG]

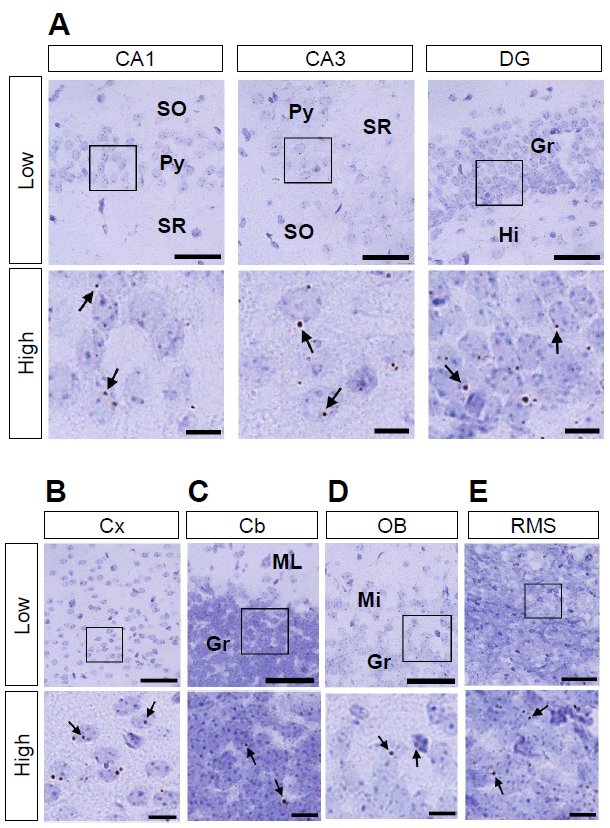

Supplement: FIGURE S2 — AhR mRNA expression in various regions of mice on PND 14. Brown punctae (arrows) represent AhR transcript in the hippocampus (A), cerebral cortex (B), cerebellum (C), olfactory bulb (D), and RMS (E). “Low” and “High” indicate magnification. High magnification images represent the areas enclosed by boxes in the low-magnification images. Scale bars = 50 and 10 μm in low and high magnification images. Gr, granule cell layer; Hi, hilus; Mi, mitral cell layer; ML, molecular layer; Py, pyramidal cell layer; SO, stratum oriens; SR, stratum radiatum. [file Image_2.JPEG]
